# Predicting a Potential Link to Antidepressant Effect: Neuroprotection of Zhi-zi-chi Decoction on Glutamate-induced Cytotoxicity in PC12 Cells

**DOI:** 10.3389/fphar.2020.625108

**Published:** 2021-01-25

**Authors:** Yin Zhang, Yusha Luo, Dongqi Zhang, Bo Pang, Jun Wen, Tingting Zhou

**Affiliations:** ^1^ School of Pharmacy, Second Military Medical University, Shanghai, China; ^2^ Shanghai Key Laboratory for Pharmaceutical Metabolite Research, School of Pharmacy, Second Military Medical University, Shanghai, China

**Keywords:** ZZCD, compatibility, antidepressant effect, PC12 cell, chronic unpredictable mild stress

## Abstract

Zhi-zi-chi Decoction (ZZCD), composed of *Fructus Gardeniae* (Zhizi in Chinese, ZZ in brief) and *Semen sojae praeparatum* (Dandouchi in Chinese, DDC in brief), has been used as a drug therapy for depression for thousands of years in China. However, the antidepressant mechanism of ZZCD still remains unknown. This study was aimed at exploring antidepressant effects of ZZCD from the aspect of neuroprotection based on herb compatibility. Glutamate-treated PC12 cells and chronic unpredictable mild stress (CUMS)-induced rats were established as models of depression *in vitro* and *in vivo* respectively. Cell viability, lactate dehydrogenase (LDH), apoptosis rate, reactive oxygen species (ROS), glutathione reductase (GR) and superoxide dismutase (SOD), and the expressions of Bax, Bcl-2 and cyclic adenosine monophosphate-response element binding protein (CREB) were measured to compare neuroprotection among single herbs and the formula *in vitro*. Behavior tests were conducted to validate antidepressant effects of ZZCD *in vivo*. Results showed that the compatibility of ZZ and DDC increased cell viability and activities of GR and SOD, and decreased the levels of LDH, apoptosis cells and ROS. Besides, the expressions of Bcl-2 and CREB were up-regulated while that of Bax was down-regulated by ZZCD. Furthermore, the compatibility of ZZ and DDC reversed abnormal behaviors in CUMS-induced rats and displayed higher efficacy than any of the single herbs. This study revealed that the antidepressant effects of ZZCD were closely associated with neuroprotection and elucidated synergistic effects of the compatibility of ZZ and DDC based on it.

## Introduction

Depression is characterized by sadness, loss of interest or pleasure, feelings of guilt or low self-worth, and other symptoms. As a common mental disorder worldwide, depression is a leading cause of disability and a major factor of the overall global burden of disease. According to the World Health Organization (World Health Organization, 2017), over 300 million people suffer from depression and nearly 800 thousand of them die due to suicide every year. However, only fewer than half of those depressant patients receive antidepressant treatment (World Health Organization, 2017). Although plenty of antidepressant drug have been developed including monoamine oxidase inhibitors, tricyclic antidepressants, and selective serotonin reuptake inhibitors (Hillhouse and Porter, 2015), treatment-resistant depression still exacts a substantial toll on quality of life in patients with depression (Mrazek et al., 2014). Thus, increasing researchers pay more attention on the mechanisms of depression and drugs with potential therapeutic targets such as traditional Chinese medicine (TCM).

Considering the complicated pathogenesis of diseases and the characteristics of multi-components and multi-targets of TCM, increasing western researchers are accepting TCM formulas which have been used for thousands of years in China (Li et al., 2020). Compatibility plays a crucial role in TCM theory, which requires TCM formulas to meet the rule of “monarch, minister, assistant, and guide” (Zhou et al., 2016). In TCM formulas, compatibility theoretically means herb-herb interactions which occur at different levels including pharmaceutics, pharmacokinetics (PK), and pharmacodynamics (PD) (Zhou et al., 2016). From the aspect of PD, the compatibility of herbs can enhance drug efficacy, decrease toxicity, or produce new pharmacological effects that does not exist in any single herbs. Composed of dried ripe fruits of *Gardenia jasminoides* J. Ellis (genus *Gardenia*, family *Rubiaceae*, *Fructus Gardeniae*, Zhizi in Chinese, ZZ in brief) and fermented ripe seeds of *Glycine max* (L.) Merr. (genus *Glycine*, family Leguminosae, *Semen sojae praeparatum*, Dandouchi in Chinese, DDC in brief), Zhi-zi-chi decoction (ZZCD) is a typical TCM herb pair (ZZ is the monarch and DDC is the minister) chronicled in Shang Han Lun, which has been frequently utilized for treatment of depression, febrile diseases and agrypnia since approximate 202 AD in China (Yue et al., 2010) Studies have reported that iridoid glycosides and isoflavones are major active ingredients of ZZCD that showed various activities such as anti-inflammation (Koo et al., 2006), antipyretic effect (Debnath et al., 2011) and anti-tumor effects (Hsu et al., 1997). Geniposide, one of the iridoid glycosides of ZZCD, exerts antidepressant effects on chronic unpredictable mild stress (CUMS)-induced depressive rats (Cai et al., 2015). Moreover, geniposide can pass through blood brain barrier, distribute targetedly in the brain (Qu et al., 2014), which suggests a potential link between ZZCD and its antidepressant effects. Chemical characterizations of single herbs and the formula ZZCD have been investigated qualitatively and quantitatively in our previous work (Han et al., 2015; Guo et al., 2018; Zhang et al., 2019). However, antidepressant effects of ZZCD are seldom reported neither *in vivo* nor *in vitro* with the mechanism involved remaining unknown, which limits our incisive and comprehensive understanding of it.

A majority of current antidepressants are designed based on the monoamine hypothesis, the most commonly proposed mechanism of depression. However, the emergence of therapeutic delay becomes a major obstruction in the application of these antidepressants (Pham and Gardier, 2019). In addition, approximate 30% patients with depression show resistance or non-response to these antidepressants (Mrazek et al., 2014). Thus, increasing antidepressant researches have concentrated on the glutamate system, another factor that contributes to the pathogenesis of depression (Murrough et al., 2017). Neuroimaging and post-mortem studies reported excessively elevated glutamate levels in the plasma, cerebrospinal fluid, and brains of patients with depression (Zarate et al., 2008). Moreover, abnormal glutamate release and glutamate receptor activity potentially contribute to neuron loss and the activation of cellular apoptosis, leading to neuronal atrophy in the prefrontal cortex and hippocampus (Duman et al., 2016). Based on this hypothesis, several cell models of depression have been established for antidepressant research. Similar to the phenotype of sympathetic neurons, the PC12 cell is an applicable model system for neurobiological and neurochemical studies (Greene and Tischler, 1976). In particular, glutamate-induced PC12 cells were a common cell model of depression and the neuroprotection against glutamate-induced neurotoxicity was associated with antidepressant effects (Mao et al., 2010; Xie et al., 2013).

Oxidative stress is believed to be associated with the advancement of depression by the study of oxidative markers (Yusuf et al., 2018), including reactive oxygen species (ROS) and intrinsic antioxidant enzyme (Hritcu et al., 2017), which were reported with abnormal changes in plenty of literatures. In addition, neuron damages and apoptosis following oxidative stress in return further accelerate the depression process. Cyclic adenosine monophosphate (cAMP)-response element binding protein (CREB) that participates in the cAMP-PKA-CREB-BDNF pathway is claimed to be closely related to depression (West et al., 2001). And the phosphorylation of CREB activates effector proteins and is essential for neuronal survival (Alberini, 2009).

In this study, the compatibility of ZZCD was investigated to reveal its antidepressant effects from the aspect of neuroprotection in glutamate-induced PC12 cells. In addition, the behavior tests upon a CUMS-induced rat model were conducted to validate the antidepressant effects of ZZCD. Our study comprehensively explored the antidepressant effects of ZZCD both *in vitro* and *in vivo* and the synergistic effects of ZZ and DDC compatibility, results of which may provide as references for future study of TCM-based antidepressants and compatibility.

## Materials and Methods

### Material

Dried ripe fruits of *Gardenia jasminoides* J. Ellis (*Fructus Gardeniae*) (No. 180525) and the fermented ripe seeds of *Glycine max* (L.) Merr. (*Semen sojae praeparatum*) (No. 180716-1) were purchased from Tong Han Chun Tang Chinese Herbal Factory (Shanghai, Chinese). Authenticated by Professor Lu-Ping Qin, *Fructus Gardeniae* (Voucher number 2018082001) and *Semen sojae praeparatum* (Voucher number 2018082002) were deposited at the herbarium of pharmaceutical analysis, School of Pharmacy, Second Military Medical University, Shanghai, China. Genipin-1-β-D-gentiobioside (wkq18010504) was obtained from Weikeqi (China); Geniposide (No. 1203A024) from Solarbio (China); Daidzin (23270025) and Genistin (26210050) from ANPEE (China); Glycitin (AF7042812) and Daidzein (AF7041801) from ALFA (China).

PC12 cells (No. KCB93033YJ) were obtained from Cell Bank of the Chinese Academy of Sciences (Kunming, China); DMEM-High glucose from Hyclone (United States); 0.25% trypsin-EDTA (1x) from Gibco (United States); Fetal bovine serum (FBS) from Biological Industries (Israel); Glutamate, Dimethylsulfoxide (DMSO), and 3-(4,5-dimethyl-thiahiazol-2-thiazolyl)-2,5-diphenyl-2H- tetraxoliumbromide (MTT) from Sigma (United States); Fluoxetine from Yuanye (China). Cytotoxicity LDH Assay kit from Dojindo (China); The Annexin V FITC/Propidium iodide apoptosis detection kit from MultiSciences (China); ROS, GR, and SOD Assay Kit from Beyotime (China); Bicinchoninic acid (BCA) protein assay kit from Bio-Rad (United States). Deionized water was collected by a laboratory water purification system (HITECH Instruments CO., LTD). BCA protein detection kit was purchased from Thermo Scientific (United States). SDS-PAGE electrophoresis buffer powder, transfer membrane buffer powder, TBS buffer powder, primary antibody diluent, and skim milk were obtained from Servicebio (China). Rabbit monoclonal antibody CREB, Bax, rabbit polyclonal antibody bcl-2, β-tubulin, and fluorescent labeled goat anti-rabbit IgG were purchased from Abcam (China). Sodium carboxymethyl cellulose (CMC-Na) and sucrose were obtained from Sangon Biotech Co., Ltd. (Shanghai).

### Preparation of ZZ, DDC, and ZZCD Extract

ZZ and DDC were powdered and sieved respectively. Then three groups of raw herbs were prepared including ZZ, DDC, and ZZCD (ZZ:DDC = 1:1, w/w). 100 g powder of each group was extracted twice by refluxing with 800 ml 50% ethanol for 1 h. Then the filtrate was mixed and centrifuged for 10 min at 3,000 r/min. After the supernatant was collected, the crude extract was prepared by evaporating to dryness by rotary vaporization at 60 °C. Then the crude extract was dissolved and loaded on D101 macroporous adsorption resins for 2 h. Different concentration of ethanol (0, 10%, 20%, 30%, 40%) was used to wash resin. Then only the 40% ethanol eluent was collected, concentrated by evaporation, and dried to extract powder. For cell experiments, the extract powder of ZZ, DDC, and ZZCD were weighed and dissolved in fresh culture medium to a concentration of 100 mg/ml.

### UPLC-Q-TOF/MS Analysis

The qualitative analysis of ZZCD was performed on Agilent 6538 UHD Accurate-Mass Q-TOF LC/MS system. XSelect HSS T3 column (100 × 2.1 mm, 2.5 μm) was used at 40 °C and the flow rate was 0.4 ml/min. The mobile phase consist of A (water-formic acid, 100:0.1, v/v) and B (acetonitrile-formic acid, 100:0.1, v/v) in a liner gradient program: 0–2 min, 5%B; 2–17 min, 2%-98%B; 17–19 min, 98%B. ZZCD was dissolved in 50% methanol at 50 mg/ml equivalent to raw herbs, and the inject volume was 3 μL. Chromatography was acquired in both positive and negative ion modes with a mass range from 50 to 1,500 Da.

### HPLC Analysis

The quantitative analysis of ZZCD was performed on a Shimadzu HPLC system. ZZCD was dissolved in 50% methanol at 100 mg/ml equivalent to raw herbs. The sample was separated on a XTerra C18 column (250 × 4.6 mm, 5 μm) at 25 °C and with a linear gradient of water-acetic acid (100:0.1, v/v, A) and acetonitrile (B) as follows: 0–30 min, 10%-20%B; 30–50 min, 20%-40%B; 50–51 min, 40%-10%B; 51–60 min, 10%B. The flow rate was 1 ml/min, the detection wavelength was 254 nm, and the inject volume was 10 μL. External standard method was used for compound quantitative analysis.

### Method Validation of HPLC Analysis

The specificity of the method was evaluated by analyzing blank solution (50% methanol), pooled standards, and ZZCD. Linearity was assessed by analyzing the standard curve using six different concentrations. The calibration curve was constructed on a concentration range of 34.47–1733 μg/ml for genipin-1-β-D-gentiobioside, 100.0–5,000 μg/ml for geniposide, 0.8667–43.33 μg/ml for daidzin, 0.2667–13.33 μg/ml for glycitin, 0.8667–43.33 μg/ml for genistin, 0.6667–33.33 μg/ml for daidzein. The precision of the method was evaluated by analyzing one pooled standard for 6 times. The repeatability of the method was assessed by analyzing six parallel pooled standards. One pooled standard was injected into HPLC system at 0, 1, 2, 4, 8, 12, 24 h to determine the stability of the solution. And the relative standard deviation of peak areas of six compounds was calculated. An appropriate amount of ZZCD was divided into one portion as the control group, and the other portion spiked with marked standards at approximate concentration as ZZCD. After HPLC analysis, recovery was calculated using following equation: recovery (%) = (total amount detected–amount original)/amount spiked × 100.

### Cell Culture

Kept in a 5% CO_2_ incubator at 37 °C, PC12 cells were cultured in DMEM-High glucose medium, supplemented with 10% FBS, 100 μg/ml streptomycin, and 100 U/ml penicillin. Cells were seeded in 55 cm^2^ culture dishes at a density of 5×10^4^ cells/ml and preserved to passage every 2 days. Only exponentially growing cells were used in the following experiments.

### Cell Viability Assay

The effects of glutamate and extracts of ZZ, DDC, and ZZCD on PC12 cells were determined by MTT method. Briefly, cells were seeded at a density of 5×10^3^ cells/well in 96-well plates and kept for 24 h in incubator. After incubated with a series of concentration of glutamate and extracts, cells were incubated for another 24 h. Then the medium was removed and each well was washed twice with PBS before added with 100 ml fresh medium and 20 µL MTT (5 mg/ml). After another 4 h of incubation, removed the turbid medium and added 150 µL DMSO to each well to dissolved the formazan crystals. The optical density (OD) values were measured at 570 nm by microplate reader. The cell viability was calculated as follows: cell viability (%) = (OD of treated cells/OD of control cells) × 100.

### Lactate Dehydrogenase Release Assay

The release of LDH into the culture by dead or damaged PC12 cells was determined by LDH Assay kit. After PC12 cells was treated with glutamate and other extracts, 100 µL culture medium was collected in a new 96-well plate and incubated with 100 µL working solution for 30 min according to the protocol. Then 50 µL stop solution was added and the OD values were measured at 490 nm by microplate reader immediately.

### Apoptosis Assay

To detect apoptosis of PC12 cells, the Annexin V FITC/Propidium iodide (PI) apoptosis detection kit was performed. PC12 cells were seeded in 6-well plate at a density of 1×10^5^ cells/well. After different treatment, cells were harvested and stained with Annexin V FITC and PI for 15 min at room temperature. Then the percentage of apoptotic cells was measured using flow cytometer.

### Assessment of Reactive Oxygen Species

The DCFH-DA fluorescent probe was used to detect the level of intracellular ROS. After PC12 cells were treated with glutamate in the presence or absence of other extracts for 24 h, cells were harvested and stained with DCFH-DA solution for 20 min at 37 °C. Subsequently, the cells were washed three times with fresh culture medium to remove excess probe. The ROS level was determined by measuring the fluorescence intensity of DCF using flow cytometer.

### Detection of Glutathione Reductase and Superoxide Dismutase Assay

The detection of GR and SOD was performed simultaneously. After PC12 cells were treated with glutamate and other extracts, harvested cells were lyzed on ice for 30 min. Then centrifuged the sample under 4 °C at 13,000 rpm for 15 min and collected the supernatant. Subsequently, the BCA protein assay kit (Bio-Rad) was used to measure protein concentration and the GR and SOD assay kit was preformed to determine the activities of glutathione reductase and superoxide dismutase.

### Western Blot Analysis

PC12 cells in logarithmic growth stage were inoculated in 6-well plates at the density of 5×10^4^ cells/mL, incubated at 37 °C and 5% CO_2_ for 24 h. After treated with Glu and different concentration of ZZCD, cells were incubated for another 24 h and then the medium was discarded. Afterward the plates were washed 3 times with PBS. Cell lysis buffer was added to each well and the cells were splitted under 4 °C for 30 min. After that, cells were scraped down gently and collected into 1.5 ml centrifuge tube. Cell debris were centrifuged at 4 °C for 10 min at 10,000 g. The supernatant was taken for protein concentration detection with BCA kit and each sample was normalized into the same concentration with PBS. Protein loading buffer was added and the protein samples were degenerated in 100 °C water bath for 10 min. The electrophoresis process was undertaken with 12% SDS-PAGE gel. PVDF membrane was used for transferring and 5% skim milk was used for blocking for 90 min. CREB (1:1,000), Bcl-2 (1:2,000), Bax (1:10,000) and β-tubulin (1:500) antibodies were used for incubation overnight at 4 °C. After washing with TBST for 10 min × 3 times, goat anti rabbit IgG was used as secondary antibody for incubation for 90 min, after which the films were washed with TBST again. Odessey infrared fluorescence scanning imaging system was used for development. ImageJ software was used to determine the gray value of the target protein and β-tubulin to obtain the relative gray value of the target protein.

### Animals and Drug Administration

All animal experiments were approved by the Ethics Committee of the Second Military Medical University (Approval number 2019022601) (Shanghai, China). A total of 48 of male Sprague-Dawley rats (weighing 180–220 g) were obtained from Shanghai Sippr-BK laboratory animal Co. Ltd. (SCXK2013-0016). Under appropriate temperature (22 ± 2 °C) and humidity (55 ± 5%), animals were adapted to the new experimental environment (12 h light dark cycle) for 1 week. After that, the rats were divided randomly into six groups with eight rats in each group, including control (CON), model (CUMS), Zhi-zi-chi decoction (ZZCD), *Gardeniae Fructus* (ZZ), *Semen sojae praeparatum* (DDC), and Fluoxetine (Flu). The Flu group served as the positive group. For the next 7 weeks (Zhao et al., 2020), all the rats were fed alone following the CUMS design except CON group (Tables 1, 2). For the last 2 weeks, the rats in ZZCD, ZZ, and DDC group were gavaged with ZZCD, ZZ, and DDC at a dosage of 5 g/kg (converted into raw material) once a day respectively. In addition, the rats in Flu group were administered with 10 mg/kg fluoxetine (Jiang et al., 2020), and the rats in CON and CUMS group were treated with CMC-Na solution. ZZCD, ZZ, DDC and fluoxetine were dissolved in 0.5% CMC-Na solution.

**TABLE 1:** The description of different stress in CUMS design.

No	Stress	Description
1	Damp bedding	damp bedding for 24 h
2	Cage tilting	Cage tilting (45°) for 24 h
3	Day–night reversal	Reversal of light/dark cycle for 24 h
4	Food deprivation	Food deprivation for 24 h
5	Water deprivation	Water deprivation for 24 h
6	Restraint	Forced physical restraint for 2 h
7	Swimming	Swimming for 6 min in 4 °C cold water
8	Horizontal oscillation	Horizontal oscillation for 5 min
9	Tail suspension	Tail suspension for 6 min

**TABLE 2:** The CUMS design in detail.

Time	Day 1	Day 2	Day 3	Day 4	Day 5	Day 6	Day 7
1st week	Cage tilting	Restraint	Swimming	Tail suspension	Damp bedding	Horizontal oscillation	Food deprivation
2nd week	Cage tilting	Day–night reversal	Food deprivation	Horizontal oscillation	Water deprivation	Swimming	damp bedding
3rd week	Restraint	Cage tilting	Horizontal oscillation	Swimming	damp bedding	Tail suspension	Food deprivation
4th week	Day–night reversal	Swimming	Horizontal oscillation	Tail suspension	damp bedding	Cage tilting	Food deprivation
5th week	damp bedding	Cage tilting	Horizontal oscillation	Tail suspension	Swimming	Restraint	Cage tilting
6th week	Day–night reversal	damp bedding	Swimming	Horizontal oscillation	Cage tilting	Tail suspension	Water deprivation
7th week	damp bedding	Swimming	Tail suspension	Horizontal oscillation	Cage tilting	Restraint	Food deprivation

### Sucrose Preference Test

The sucrose preference test (SPT) was conduct on day 0, 22, 32, and 50. Before SPT, the rats were given two bottles of 1% sucrose solution for 24 h, and then one of the bottles was replaced by water for another 24 h. In SPT, all rats were deprived of food and water for 23 h. After that, the rats were given a free choice of two bottles of liquid (one with 1% sucrose solution, the other with water) for 1 h. The weight of the consumed sucrose solution and water was measured to calculate the sucrose preference rate (Willner et al., 1987). The sucrose preference rate was calculated with the following formula:

Sucrose preference rate(%)=consumed sucrose solution/(consumed sucrose solution+consumed water).

### Forced Swim Test

The forced swim test (FST) was conduct on day 0, 23, 33, and 51. The rats were separately placed in a cylinder (60 cm in height and 40 cm in diameter) filled with water at 25 ± 2 °C to a depth of 40 cm. Each rat was adapted for 2 min and the total immobility time in the following 4 min was recorded. The immobility time was described as the amount of time that the rats spent keeping their heads above the water without struggling (Porsolt et al., 1977).

### Tail Suspension Test

The tail suspension test (TST) was conduct on day 0, 25, 32, and 52. With their heads 10 cm above the ground, the rats were suspended by their tails with adhesive tape from a ledge. The whole test lasted 6 min. Each rat was adapted for 2 min then the total immobility time in the remaining 4 min was recorded. The immobility time was defined as the amount of time that rats were suspended passively and remained completely motionless (Steru et al., 1985).

### Statistical Analysis

Expressed as mean ± standard deviation (SD), experimental data were analyzed using One-way analysis of variance (ANOVA) text on Graphpad Prism (GraphPad Software, United States). Statistically significant difference was set at *p* < 0.05 and each experiment was repeated three times independently.

## Results

### Validation Results of HPLC Analysis

The results of method validation were shown in Table 3. All correlation coefficients of six regression equation exceeded 0.999, indicating good linearity of the calibration curves. In addition, the precision ranged from 1.1 to 2.1%, the repeatability ranged from 0.3 to 3.7%, the stability ranged from 1.5 to 4.2%, and the recovery ranged from 1.0 to 2.9%. The results showed that all the values were within the acceptance criteria and the method of HPLC analysis was capable for quantification of these six compounds.

**TABLE 3:** The results of method validation.

Compound	Regression equation	*R* ^2^	Precision (%)	Repeatability (%)	Stability (%)	Recovery (%)
Genipin-1-β-D-gentiobioside	y = 5,445.1x + 10,175	0.9999	1.7	2.0	1.3	1.5
Geniposide	y = 7,891.5x + 289,745	0.9995	1.2	0.3	0.8	1.0
Daidzin	y = 22,070x − 2,437.4	0.9999	1.3	0.3	0.5	1.5
Glycitin	y = 32,742x − 3,296.5	0.9999	1.1	1.8	3.6	1.9
Genistin	y = 36,003x − 7,412.6	0.9999	1.3	1.4	0.9	2.9
Daidzein	y = 35,387x − 1,998.9	0.9999	2.1	3.7	4.2	1.3

### Chemical Characterization Analysis

**Figure 1:**
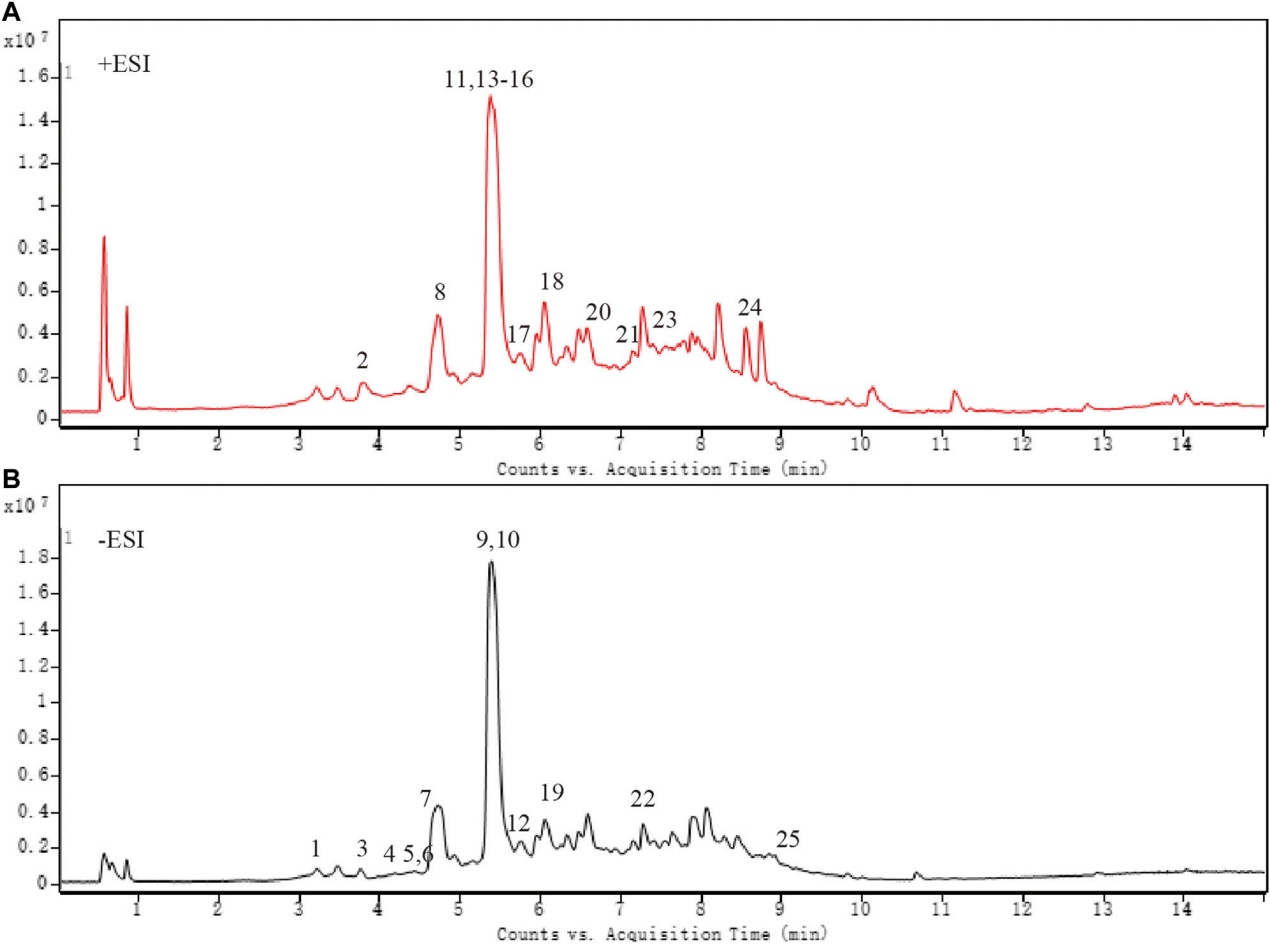
Total ion chromatogram of ZZCD in positive ion mode **(A)** and negative ion mode **(B)**.

**Figure 2:**
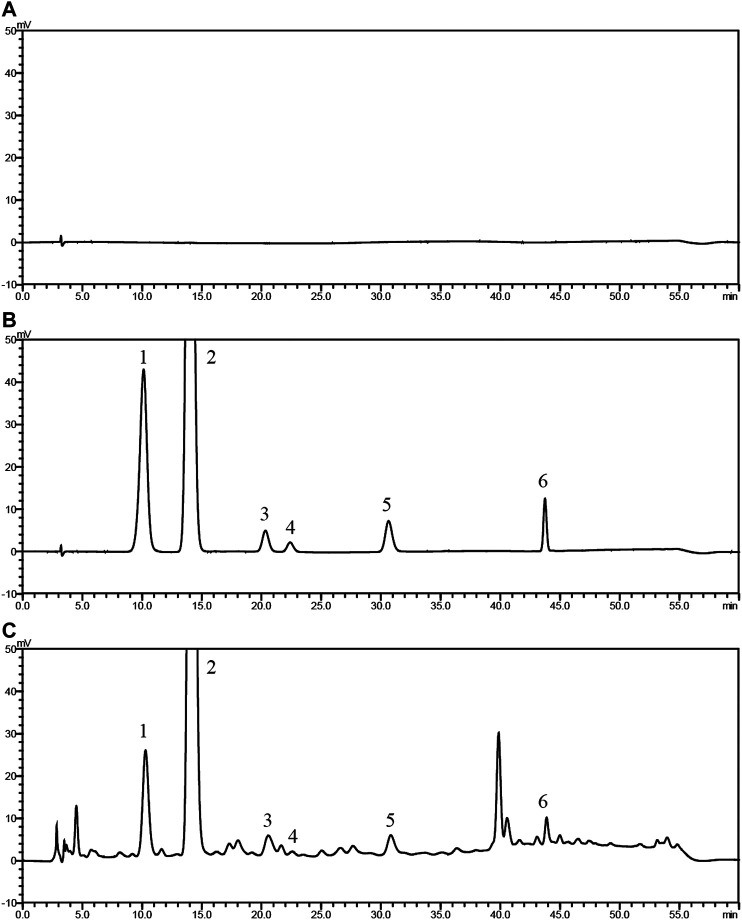
HPLC chromatograms of 50% methanol **(A)**, pooled standards **(B)**, and ZZCD **(C)**. Peak 1 to 6: Genipin-1-β-D-gentiobioside; Geniposide, Daidzin, Glycitin, Genistin, and Daidzein.

As shown in Figure 1 and Table 4, 25 substances in ZZCD were identified by comparing the mass spectrum and fragment information with literature. Among them, six key compounds were quantified by their calibration curves including Genipin-1-β-D-gentiobioside, Geniposide, Daidzin, Glycitin, Genistin, and Daidzein (Figure 2; Table 5). These six compounds were believed to play pivotal roles in the antidepressant effects of ZZCD for the following reasons. First and foremost, the six compounds had relatively higher abundances than other identified compounds, which is prerequisite in exerting therapeutic effects. Second, according to our previous studies of quality control of ZZ and DDC based on their fingerprints and approaches of chemometrics and statistics (Han et al., 2015; Guo et al., 2018), they made big differences in defining the quality of the two herbs. Third, our *in vitro* study conducted previously revealed that the six components had high bioactive efficacy in the antidepressant model. Taking the considerations together, the six components in ZZCD were identified to be important in exerting antidepressant effects of ZZCD.

**TABLE 4:** MS date for characterization of compounds in ZZCD.

NO.	Formula	t_R_(min)	Molecular weight	MS(+)	MS(-)	Compound identification
1	C_16_H_22_O_11_	3.366	390.1189		389.1114[M-H]^-^	Deacetylasperulosidic acid
2	C_16_H_22_O_10_	3.962	374.1222	357.118[M + H-H_2_O]^+^	373.1154[M-H]^-^	Gardoside
3	C_16_H_24_O_11_	3.962	392.133		373.1153[M-H-H_2_O]^-^	Shanzhiside
4	C_16_H_18_O_9_	4.194	354.0961		353.0882[M-H]^-^	3-O-Caffeoylquinic acid
5	C_18_H_26_O_13_	4.284	450.1389		449.1318[M-H]^-^	Gardenoside
6	C_23_H_22_O_11_	4.284	474.1141		501.1014[M + HCOO-H_2_O]^-^	6″-O-Acetylgenistin
7	C_24_H_22_O_12_	4.301	502.1096		501.1014[M-H]^-^	Malonyldaidzin
8	C_23_H_34_O_15_	4.905	550.1909	573.1827[M + Na]^+^	549.1831[M-H]^-^	Genipin-1-β-D-gentiobioside
9	C_17_H_24_O_10_	5.26	388.1344		433.1313[M + HCOO]^-^	Geniposide
10	C_17_H_26_O_11_	5.26	406.145		433.1313[M + HCOO-H_2_O]^-^	Shanzhiside methylester
11	C_11_H_14_O_5_	5.269	226.0843	227.0924[M + H]^+^	225.0766[M-H]^-^	Genipin
12	C_17_H_26_O_10_	5.442	390.1508		435.1513[M + HCOO]^-^	Loganin
13	C_21_H_20_O_9_	5.575	416.1117	417.1191[M + H]^+^	461.1103[M + HCOO]^-^	Daidzin
14	C_16_H_26_O_7_	5.616	330.1686	331.1782[M + H]^+^	375.1665[M + HCOO]^-^	Jasminoside A
15	C_22_H_22_O_10_	5.658	446.1199	447.1313[M + H]^+^	491.1189[M + HCOO]^-^	Glycitin
16	C_9_H_8_O_3_	5.798	164.0477	147.0443[M + H-H_2_O]^+^	163.0403[M-H]^-^	P-coumaric acid
17	C_16_H_26_O_8_	5.864	346.1624	329.1595[M + H-H_2_O]^+^	373.1499[M + HCOO-H_2_O]^-^	Jasminoside B
18	C_21_H_20_O_10_	6.22	432.109	433.1163[M + H]^+^	477.1058[M + HCOO]^-^	Genistin
19	C_24_H_22_O_13_	6.22	518.1041		545.0922[M + HCOO-H_2_O]^-^	Malonylgenistin
20	C_22_H_36_O_12_	6.824	492.2216	510.2564[M + NH_4_]^+^	537.2215[M + HCOO]^-^	Jasminoside I
21	C_21_H_34_O_11_	7.204	462.2116	485.203[M + Na]^+^	507.21[M + HCOO]^-^	Jasminoside T
22	C_15_H_10_O_4_	7.353	254.0573	255.0668[M + H]^+^	253.0502[M-H]^-^	Daidzein
23	C_16_H_12_O_5_	7.516	284.0698	285.0759[M + H]^+^		Glycitein
24	C_32_H_44_O_14_	8.627	652.2735	675.2654[M + Na]^+^	697.2723[M + HCOO]^-^	beta-d-gentiobiosyl crocetin
25	C_10_H_16_O_3_	9.09	184.1101	167.1072[M + H-H_2_O]^+^	165.0928[M-H-H_2_O]^-^	Jasminodiol

**TABLE 5:** The contents of six compounds in ZZCD.

NO	t_R_ (min)	Formula	Identified compound	Contents in ZZCD (mg/g)
1	10.12	C_23_H_34_O_15_	Genipin-1-β-D-gentiobioside	36.010 ± 0.671
2	14.01	C_17_H_24_O_10_	Geniposide	328.717 ± 0.540
3	20.33	C_21_H_20_O_9_	Daidzin	2.062 ± 0.005
4	22.39	C_22_H_22_O_10_	Glycitin	0.168 ± 0.003
5	30.64	C_21_H_20_O_10_	Genistin	1.304 ± 0.020
6	43.74	C_15_H_10_O_4_	Daidzein	1.160 ± 0.044

### Glutamate Changed the Morphology of PC12 Cells

**Figure 3:**
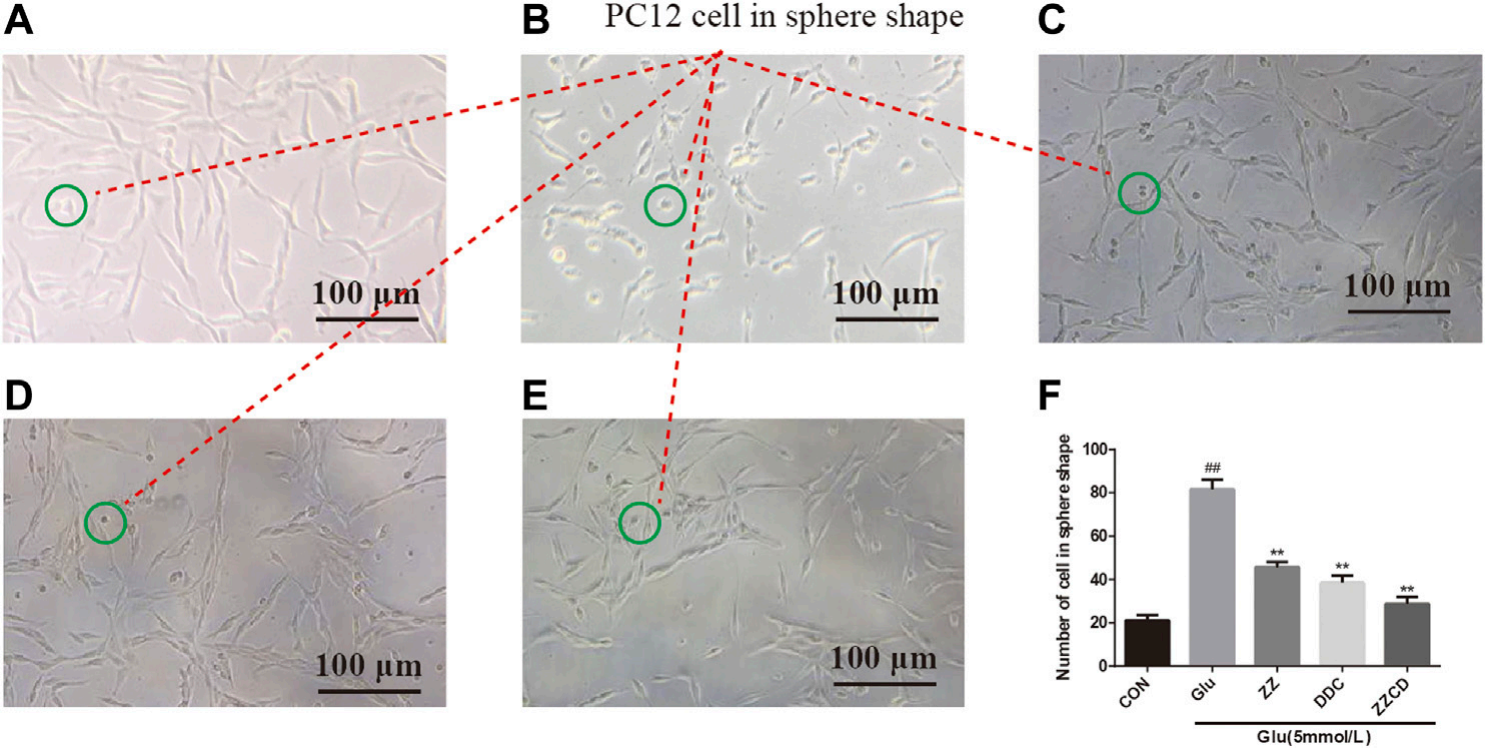
Morphology of PC12 cells in CON **(A)**, Glu **(B)**, ZZ **(C)**, DDC **(D)**, and ZZCD **(E)** group. Scale bar = 100 μm. The cell in green circle was considered as PC12 cell in sphere shape. Number of PC12 cells in sphere shape **(F)**. ^##^ *p* < 0.01 vs. control. **p < 0.01 vs. cells treated with glutamate alone (*n* = 3).

As shown in Figure 3A, PC12 cells in CON group have similar phenotype to sympathetic neurons with axons and dendrites. After stimulated by glutamate, the density of normal PC12 cells was decreased. Besides, the volume of PC12 cells decreased and the shape of them shrank into sphere (Figure 3B). The cell in the green circle was considered as PC12 cell in sphere shape. And the number of cell in sphere shape in each group was shown in Figure 3F, which could be considered as a reflection of the severity of atrophy in PC12 cells. Compared with CON group, the number of cell in sphere shape in Glu group (Figure 3B) was increased significantly, indicating glutamate cause atrophy of a large number of PC12 cells. However, ZZ (Figure 3C) and DDC (Figure 3D) treatments significantly decreased the number of cell in sphere shape. Interestingly, the most reduction of the number of cell in sphere shape in ZZCD (Figure 3E) was observed when compared with ZZ and DDC, which reflected its enhanced neuroprotective effects.

### ZZCD Inhibited Glutamate-induced Cytotoxicity of PC12 Cells

**Figure 4:**
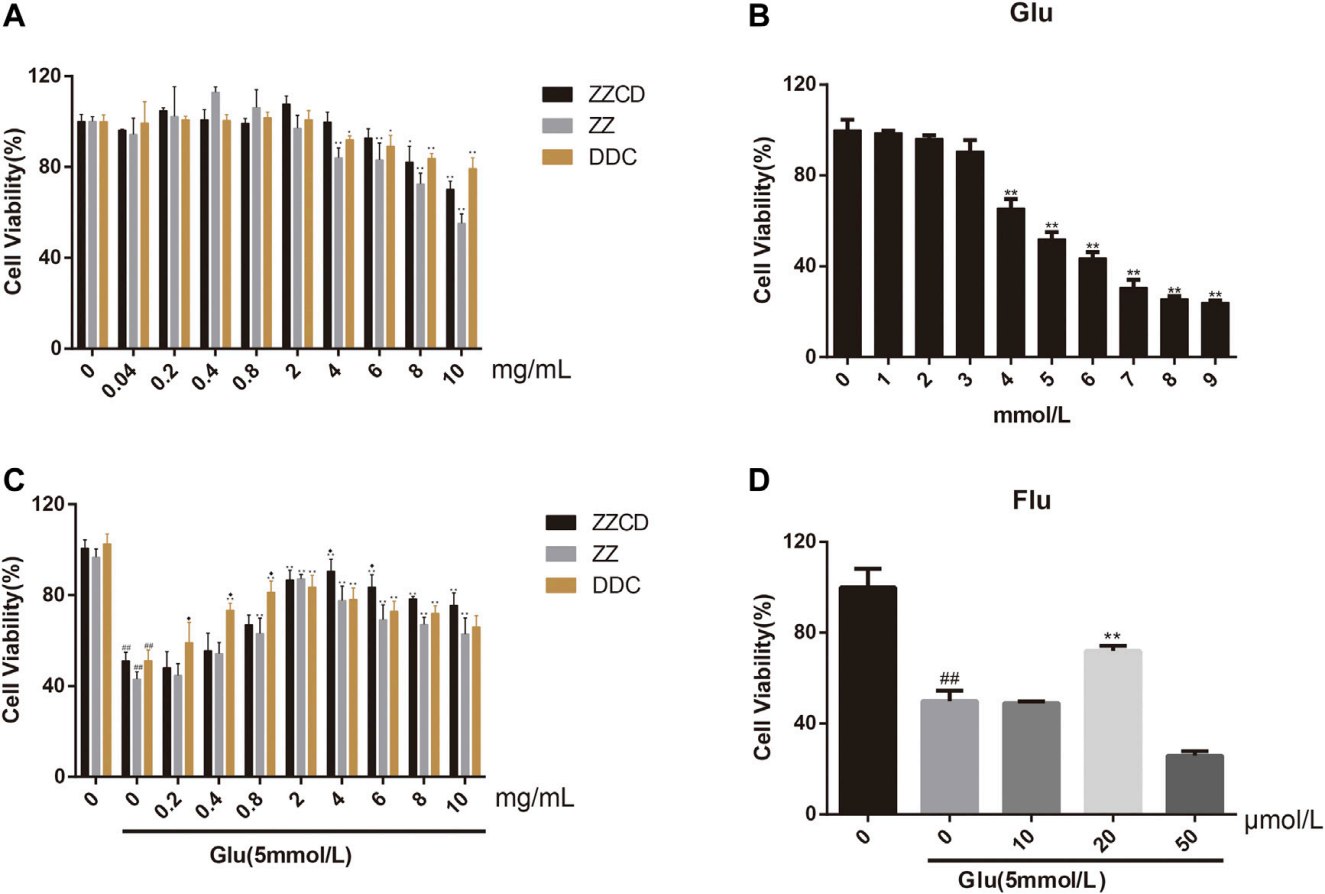
Cytotoxicity of ZZCD, ZZ, DDC **(A)**, and glutamate **(B)** in PC12 cells. Protection of ZZCD, ZZ, DDC **(C)**, and fluoxetine **(D)** in glutamate-induced cytotoxictiy in PC12 cells. ^##^ *p* < 0.01 vs. control. *p < 0.05 and **p < 0.01 vs. cells treated with glutamate alone (*n* = 3). ^♦^ *p* < 0.05 vs. cells treated with the other two group (ZZCD, ZZ, or DDC).

To explore the cytotoxicity of ZZ, DDC, and ZZCD in PC12 cells, a wide range of concentrations (0–10 mg/ml) were investigated. As shown in Figure 4, ZZCD exerted no cytotoxicity when the concentration was below 8 mg/ml, while ZZ and DDC inhibited cell proliferation when the concentration was over 4 mg/ml. The cytotoxicity of glutamate on PC12 cells was examined to obtain the optimal concentration of glutamate for subsequent experiments. Glutamate significantly suppressed cell proliferation in a dose-dependent manner from 1 to 9 mmol/L. With a cell proliferation inhibition rate of approximate 50%, 5 mmol/L glutamate was used for the following experiments. To investigate the difference of the protective effect on glutamate-induced cytotoxicity in PC12 cells among ZZ, DDC, and ZZCD, the cell viability at the same concentration was compared. DDC had significant protective effect at concentration from 0.4 to 10 mg/ml, while ZZ and ZZCD started exerting protective effect at a concentration of 0.8 mg/ml. 4 mg/ml of ZZCD exerted more profound protective effect than ZZ and DDC, which was the optimal protection concentration. As a consequence, a concentration of 4 mg/ml was chosen for further pharmacological study. In addition, 20 μmol/L fluoxetine was performed as the positive control.

### ZZCD Abolished LDH Release

**Figure 5:**
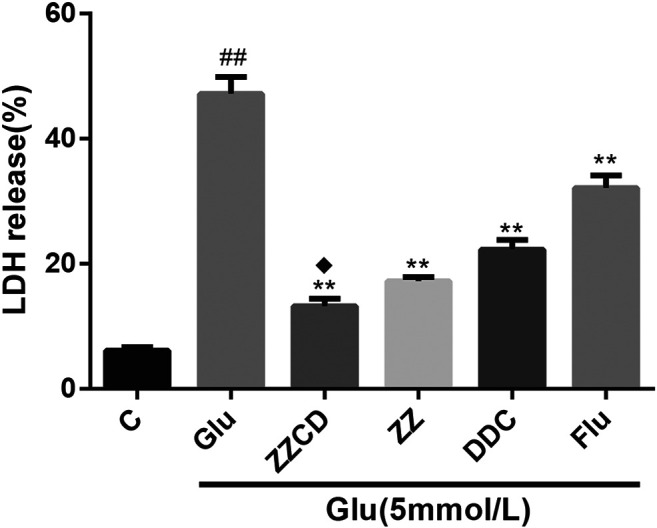
The release of LDH in glutamate treated PC12 cells. ^##^ *p* < 0.01 vs. control. **p < 0.01 vs. cells treated with glutamate alone. ^♦^ *p* < 0.05 vs. cells treated with ZZ or DDC (*n* = 3).

When cells were damaged, the destruction of mitochondria and sarcoplasmic reticulum could cause an increase in LDH levels. Compared with control group, a visible increase of LDH levels was observed in Glu group. However, as shown in Figure 5, the extracellular release of LDH was significantly decreased following treatment with ZZ, DDC, or ZZCD. Furthermore, ZZCD further decreased glutamate-induced release of LDH in PC12 cells when compared to ZZ and DDC.

### ZZCD Protected Against Glutamate-induced Apoptosis

**Figure 6:**
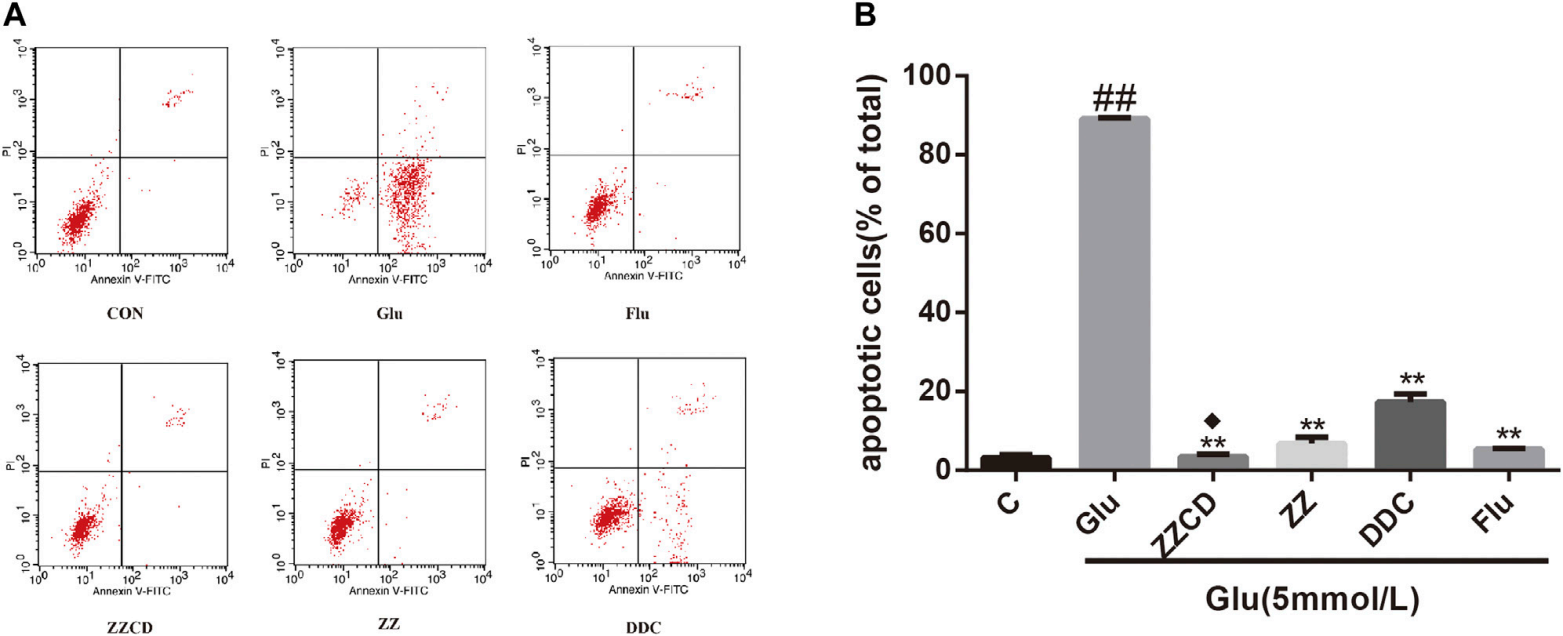
Effect of ZZCD, ZZ, and DDC on glutamate-induced apoptosis in PC12 cells using flow cytometric analysis **(A)** and quantitative analysis **(B)**. ^##^ *p* < 0.01 vs. control. **p < 0.01 vs. cells treated with glutamate alone. ^♦^ *p* < 0.05 vs. cells treated with ZZ or DDC (*n* = 3).

Since glutamate exerted cytotoxicity and destructed cell physiological stability, the assessment of apoptosis was conducted to investigate whether glutamate participated in cell apoptosis. As shown in Figure 6, the apoptotic cells as identified by Annexin V FITC and PI staining were increase significantly in Glu group when compared to control group. However, the apoptotic cells in extracts treated groups including ZZ, DDC, and ZZCD were significantly decreased in comparison with Glu group. In addition, ZZCD decreased more apoptotic cells than other extracts.

### Effect of ZZCD on ROS Production

**Figure 7:**
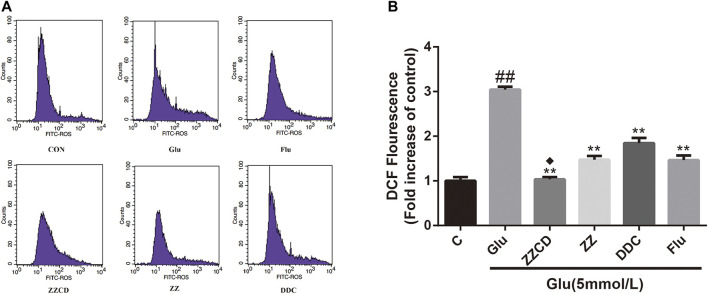
Effect of ZZCD, ZZ, and DDC on ROS production in PC12 cells treated with glutamate using flow cytometric analysis **(A)** and quantitative analysis **(B)**. ^##^ *p* < 0.01 vs. control. **p < 0.01 vs. cells treated with glutamate alone. ^♦^ *p* < 0.05 vs. cells treated with ZZ or DDC (*n* = 3).

The intracellular ROS level was determined to explore whether glutamate-induced cytotoxicity was associated with oxidative stress. Our data in Figure 7 showed the intracellular amount of ROS in Glu group was significantly increased compared with control group. However, the treatment with extracts decreased the ROS level in PC12 cells stimulated with glutamate. Furthermore, ZZCD exerted the best capability to decrease the ROS level when compared with ZZ and DDC.

### ZZCD Protected GR and SOD Activities

**Figure 8:**
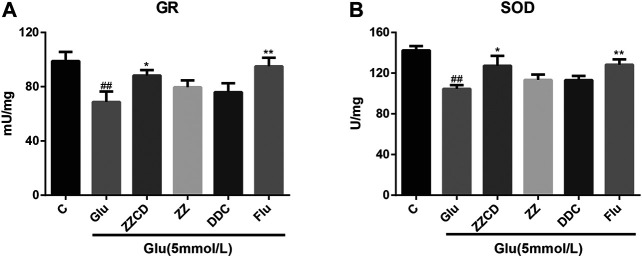
Protection of ZZCD on GR **(A)** and SOD **(B)** activities in PC12 cells treated with glutamate. ^##^ *p* < 0.01 vs. control. *p < 0.05 and **p < 0.01 vs. cells treated with glutamate alone (*n* = 3).

To assess the effect of extracts on glutamate-induced oxidative stress, the activities of antioxidant enzyme GR and SOD were estimated. As shown in Figure 8, the activities of GR and SOD were decreased in Glu group. It was evident that ZZ and DDC did not recover the GR and SOD activities while ZZCD prevented the GR and SOD activities from decreasing to some extent.

### Western Blot Analysis

**Figure 9:**
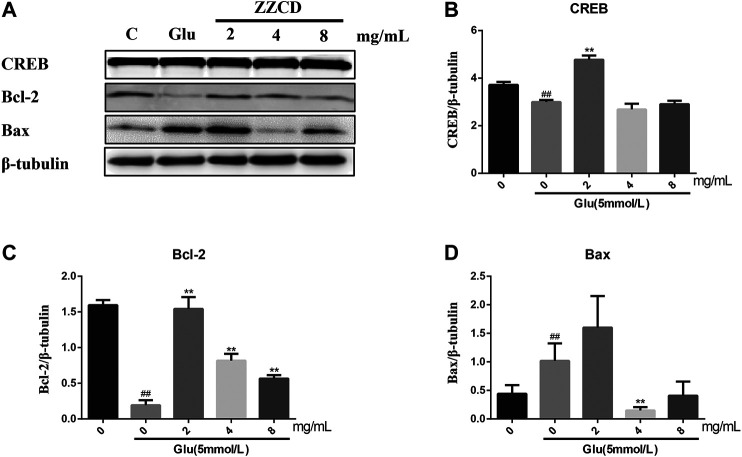
Effect of ZZCD on the expression **(A)** of CREB **(B)**, Bcl-2 **(C)**, and Bax **(D)** in PC12 cells treated with glutamate. ^##^ *p* < 0.01 vs. control. **p < 0.01 vs. cells treated with glutamate alone (*n* = 3).

Western blot results in Figure 9 show that, compared with the CON group, glutamate treatment decreases the expression level of CREB and Bcl-2, increases that of Bax. In contrast, ZZCD can reverse the above alternations, of which 2 mg/ml ZZCD acts best on CREB and Bcl-2 protein, 4 mg/ml ZZCD influences Bax most. However, 8 mg/ml ZZCD showed less effect compared with the two lower dosages.

### Effect of ZZCD on Sucrose Preference Test of CUMS Rats

**Figure 10:**
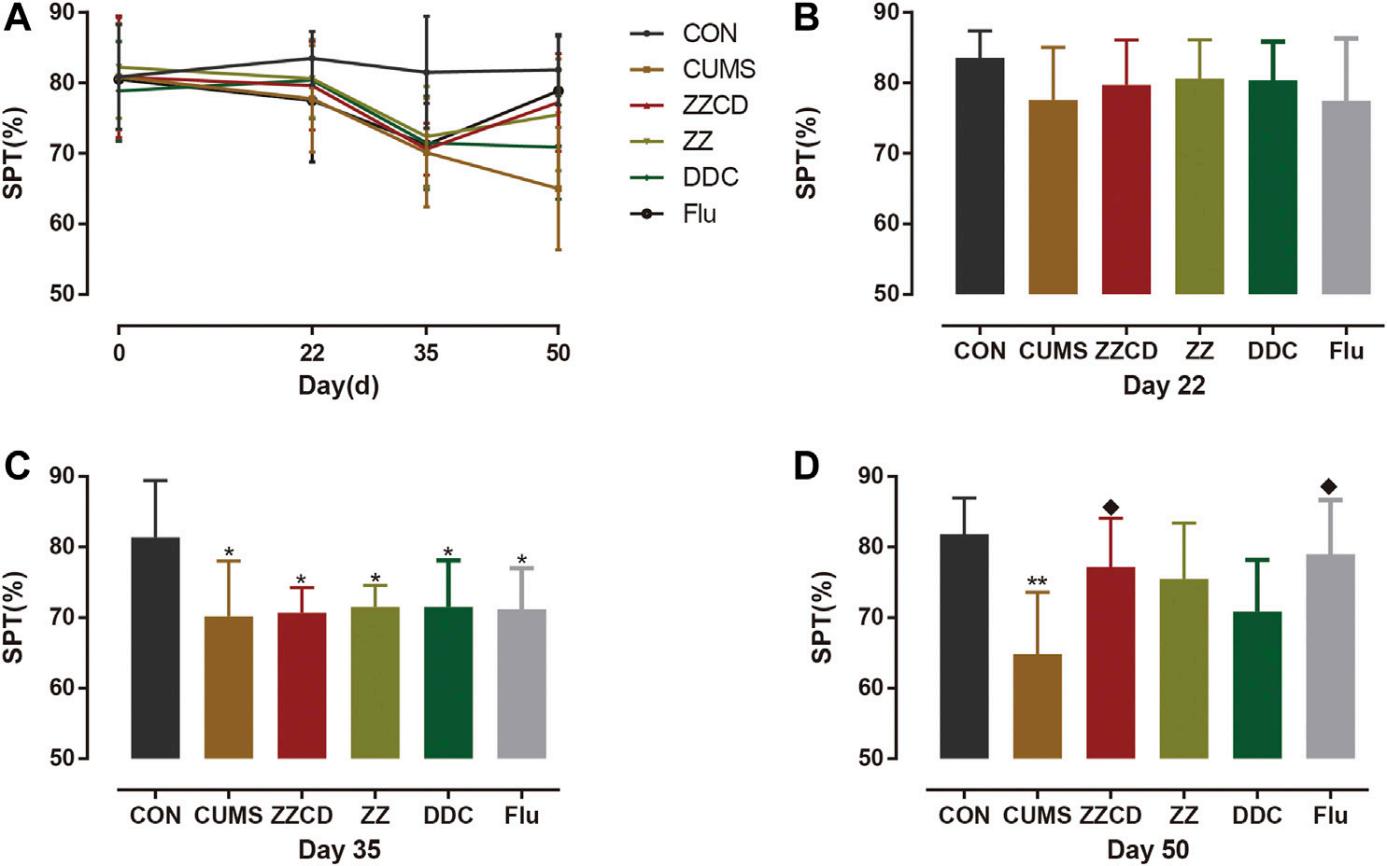
Changes in sucrose preference in six groups of rats. **(A)** Changes in sucrose preference in six groups of rats in 50 days. **(B)** Sucrose preference on day 22. **(C)** Sucrose preference on day 35. **(D)** Sucrose preference on day 50 (‾x ± *s*, *n* = 8). *p < 0.05, **p < 0.01 vs CON; ^♦^ *p* < 0.05 vs CUMS.

A core symptom of depression is anhedonia. The SPT is commonly used to evaluate anhedonia in animals. The results of SPT were shown in Figure 10. Compared with CON group, the sucrose preference rate of rats in other five groups was significantly decreased on day 35. After 2 weeks of drug administration, the sucrose preference rate of rats in ZZCD and Flu groups was significantly increased when compared with that in CUMS group on day 50. However, the sucrose preference rate of rats in ZZ and DDC did not change.

### Effect of ZZCD on Forced Swim Test of CUMS Rats

**Figure 11:**
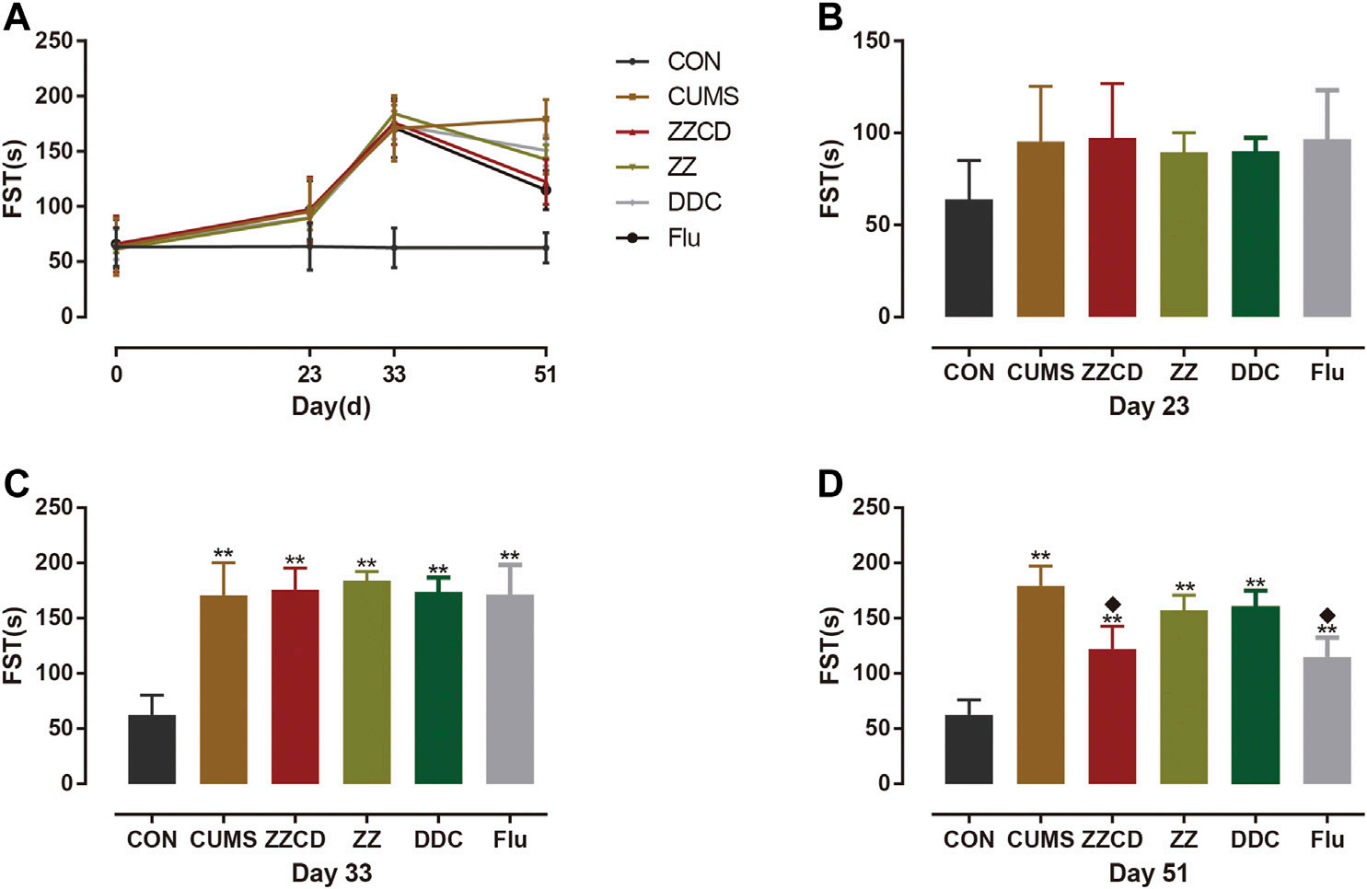
Changes in immobility time in FST in six groups of rats. **(A)** Changes in immobility time in FST in six groups of rats in 51 days. **(B)** Immobility time in FST on day 23. **(C)** Immobility time in FST on day 33. **(D)** Immobility time in FST on day 51 (‾x ± *s*, *n* = 8). **p < 0.01 vs CON; ^♦^ *p* < 0.05 vs CUMS.

The immobility in water was a form of desperate behavior and the FST was performed to evaluate the degree of desperation in animal. As shown in Figure 11, the immobility time of rats in CUMS, ZZCD, ZZ, DDC, and Flu group was increased significantly when compared with that in CON group on day 33. With 2 weeks of drug administration, the immobility time of rats in ZZCD and Flu group was decreased significantly when compared with that in CUMS group on day 51. Yet the immobility time of rats in ZZ and DDC did not change.

### Effect of ZZCD on Tail Suspension Test of CUMS Rats

**Figure 12:**
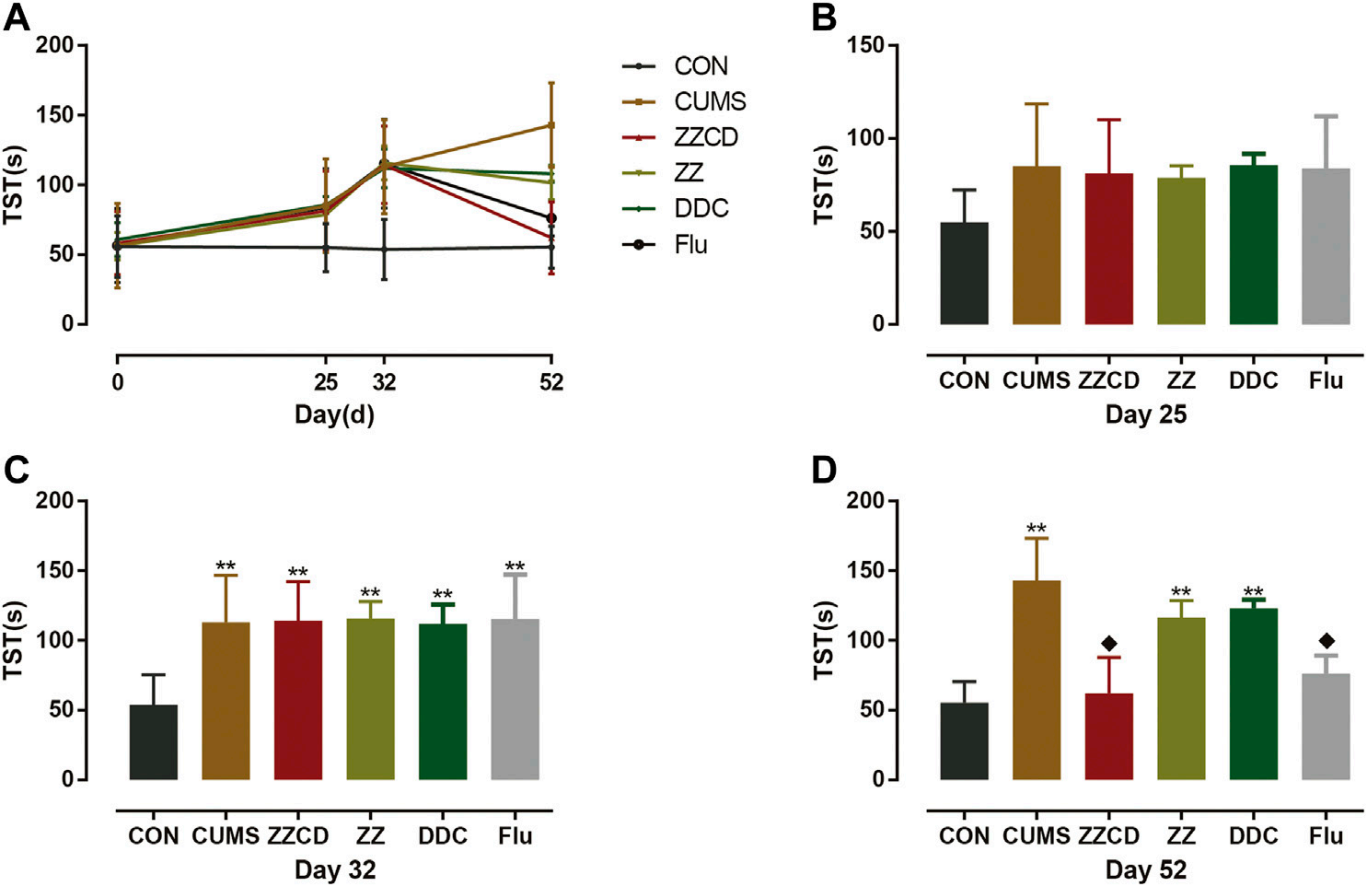
Changes in immobility time in TST in six groups of rats. **(A)** Changes in immobility time in TST in six groups of rats in 52 days. **(B)** Immobility time in TST on day 25. **(C)** Immobility time in TST on day 32. **(D)** Immobility time in TST on day 52 (‾x ± *s*, *n* = 8). **p < 0.01 vs CON; ^♦^ *p* < 0.05 vs CUMS.

The TST was suitable to detect a transition from active to passive behavior owing to unbearable environment stress, and antidepressants can reverse the immobility and stimulate escape behavior. The results of TST were concluded in Figure 12. Compared with CON group, the immobility time of rats in other groups was increased significantly on day 32. And the immobility time of rats in ZZCD and Flu group was significantly lower than that in CUMS group on day 52 while the immobility time of rats in ZZ and DDC remained the same.

## Discussion

### Enhanced Neuroprotection of Zhi-zi-chi Decoction on Glutamate-induced Cytotoxicity in PC12 Cells

Since TCM has participated in clinic therapy for thousands of years in china, a unique theoretical system has been developed for clinical application. In this theoretical system, compatibility is a crucial rule suitable for TCM formulas, which highlights reasonable and necessary interactions among different herbs including synergism (Jia et al., 2004). As a traditional herb pair (ZZ-DDC) that has been used for treating depression clinically for a long time, ZZCD is thought to comply with the rule of compatibility. Nonetheless, negligible literature reported the antidepressant effect of ZZCD. Whether the single herb ZZ or DDC is capable for antidepressant therapy also remains unknown. Thus, to figure out the significance of the compatibility of ZZCD needs to investigate the mechanism of antidepressant effects of ZZCD.

In this study, PC12 cells treated with glutamate were used to study the antidepressant effects of TCM. Extracellular administration of glutamate to PC12 cells altered the morphological characteristics of the cells, rendering them devoid of neuronal phenotype and reducing cell viability, which mimicked the atrophy of hippocampus and the decline of neurons in patients with depression (Savitz and Drevets, 2009). Here we used the concentration of glutamate when the cell proliferation inhibition rate was 50% as a model condition to study the protective effect of the TCM on glutamate-induced cell cytotoxicity more clearly. Before evaluating the efficacy of a drug, it is necessary to evaluate its safety, which also applies to TCM research. Initially extracellular administration of ZZ, DDC, and ZZCD extract to PC12 cells was performed to observe whether they are cytotoxic. As a result, within a certain concentration range, they exerted certain proliferative effects instead of inhibiting cell proliferation. Afterward, we explored the deeper meaning of compatibility by comparing the different pharmacodynamic effects of ZZ, DDC, and ZZCD at the same concentration of raw herbs. In the cell viability experiment, we found that ZZ, DDC, and ZZCD could increase cell viability and resist cytotoxicity induced by glutamate in a certain concentration range. And at some concentrations, the protective effect of the formula was better than that of the two single herbs, which suggests that the combination of ZZ and DDC enhanced the efficacy. After the optimal concentration was determined, we performed a cytotoxicity experiment, and it was observed that the formula could reduce cytotoxicity more effectively than single herbs. Both experiments clearly suggested that the combination of ZZ and DDC significantly enhanced the protective effect against glutamate-induced cytotoxicity and promoted cell proliferation. In addition, the results of apoptosis experiments showed that glutamate could cause apoptosis in PC12 cells, which ZZ, DDC, and ZZCD could inhibit. Among them, ZZCD has the strongest inhibitory effect, which also indicated that the compatibility of ZZ and DDC enhanced the inhibition of apoptosis. For oxidative stress, ROS assay results revealed a stronger capacity of ZZCD to clear oxygen radical. More surprisingly, the combination of ZZ and DDC spurred a new-drug effect that enhanced the activity of GR and SOD antioxidant enzymes. All of the above studies on the efficacy of ZZCD have demonstrated a fact that the compatibility of ZZ and DDC enhances the original pharmacodynamic effects and produces new pharmacological activity that any single herb does not have, which embodies the essence of TCM compatibility.

Our results indicated that ZZCD exerted antidepressant effects through anti-cytotoxicity, anti-oxidation, and anti-apoptosis. The dysregulation of glutamate was strongly related to the progression of depression. The levels of glutamate in serum and plasma were higher in patients with depression than those in normal people, and the severity of depressive symptoms was positively correlated with the glutamate level (Mauri et al., 1998; Mitani et al., 2006). Besides, it has been reported that impaired glutamate-glutamine cycling in the hippocampus of patients with depression leads to abnormal glutamate level (Sanacora and Banasr, 2013). Moreover, blocking this cycle by inhibiting glutamine synthase and glutamine transport could cause the depressive behavior. And the rapid antidepressant action of antidepressants was found to be associated with their enhanced stimulation of glutamate-glutamine cycling (Lee et al., 2013; Chowdhury et al., 2017). Thus the protection against glutamate-induced cytotoxicity of ZZCD indicated its antidepressant effect of anti-neurotoxicity. As for oxidative stress, unbalanced redox status has been documented in hippocampus of patients with depression (Meyer et al., 2009; Che, 2010) such as increased oxidative stress and decreased anti-oxidant capability, leading to severe DNA/RNA damage. In addition, the severity of unbalanced redox status is strongly associated with depression progression (Meyer et al., 2009; Che, 2010), which can be normalized by antidepressants (Baek et al., 2016). Moreover, increased oxidative stress and impaired anti-oxidant capability contribute to reduction of hippocampal neurogenesis and volume in depression (Lindqvist et al., 2014; Filipovic et al., 2017). As a consequence, ZZCD reversing unbalanced redox status is a crucial progress of its antidepressant effects. Neuronal apoptosis is an important contributing factor for depression and blocking neurogenesis leads to neuronal apoptosis. Pro-inflammatory cytokines can inhibit neuronal progenitor cell proliferation, and high levels of cytokines can induce neuronal apoptosis and oxidative stress (Ben-Hur et al., 2003; Iosif et al., 2006). Besides, the emerging evidence of regulation of cytokine expression and alteration of apoptosis are involved in antidepressant effect (Zhou et al., 2018; Shen et al., 2019). Therefore, the ability of ZZCD to inhibit apoptosis is closely related to antidepressant effects.

### Research on Molecular Mechanism of Antidepressant Effects of Zhi-zi-chi Decoction

**Figure 13:**
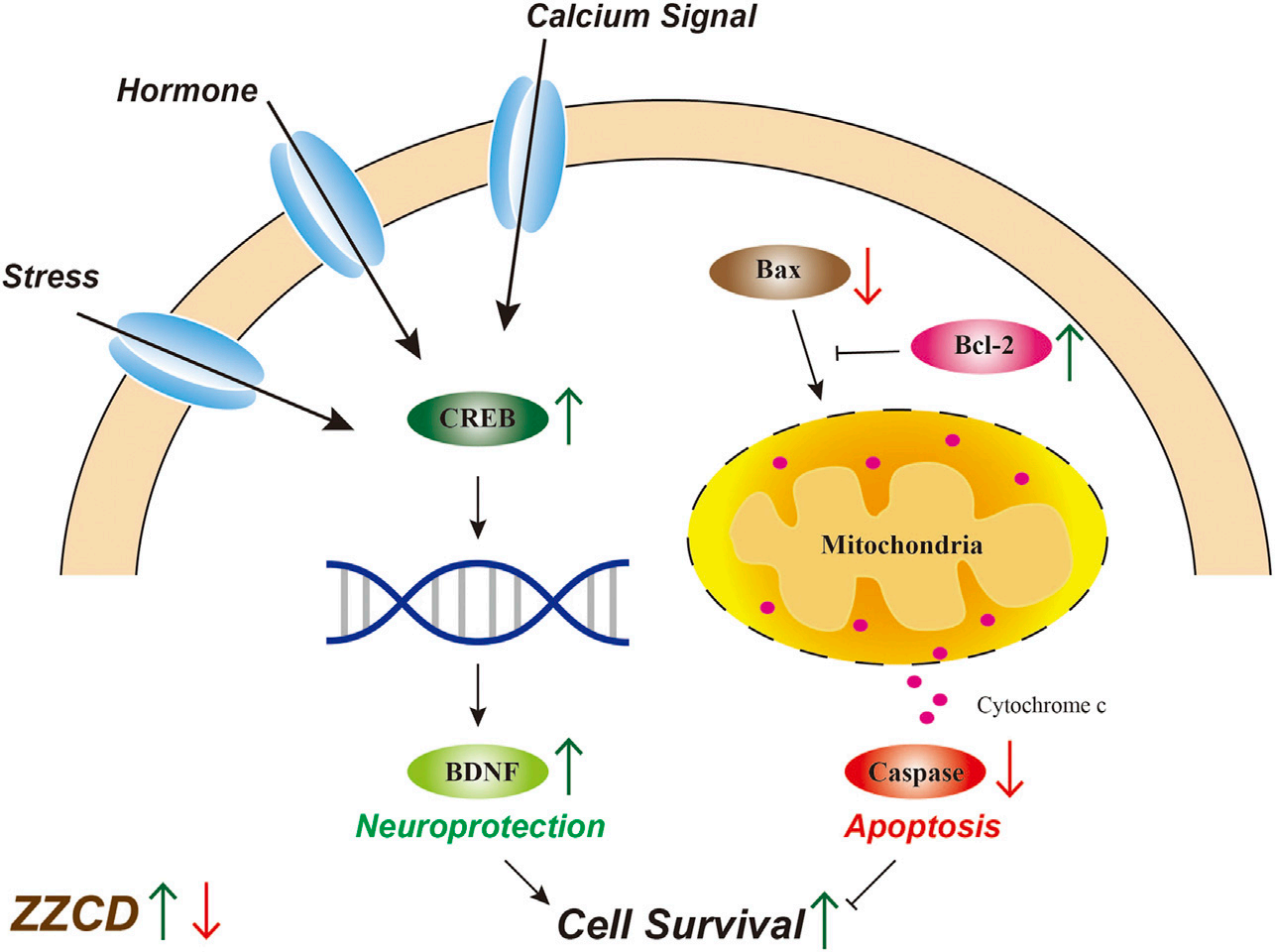
Molecular mechanism of antidepressant effects of Zhi-zi-chi decoction.

After determining the pharmacodynamic effects of ZZCD, we investigated the molecular mechanism of antidepressant effects of ZZCD. Reduced hippocampal volume has been observed in patients with depression while antidepressant treatment has been reported to reverse this decrease and promote cell survival (Videbech and Ravnkilde, 2004; Mahar et al., 2014), which suggests that the quantity and condition of viable hippocampal cells is crucial for the pathology of depression. Maintaining a healthy cell level by promoting neurogenesis may benefit the treatment of depression. Cellular death pathways contain necrotic process and apoptosis. Only in the most extreme cases, glutamate induced oxidative stress and disturbed calcium homeostasis cause hippocampal neuronal necrosis (Sapolsky, 2000). Compared with necrosis, apoptosis is programmed and controlled by apoptosis-related proteins (Yuan and Yankner, 2000). Bcl-2 is an anti-apoptotic protein that promotes neurogenesis and axon regeneration (Chen et al., 1997) while Bax is a pro-apoptotic molecule that induces apoptosis via caspase activation and proteolysis (Finucane et al., 1999). The decrease in Bcl-2 expression and increase in Bax expression observed in Glu group indicated the apoptosis in PC12 cells, which was consistent with apoptosis results. Furthermore, activated apoptotic pathway could stimulate the release of cytochrome c, followed by the activation of caspases that ultimately cause cell death (Green and Tait, 2010). In addition, our result that ZZCD restored glutamate-induced decrease in Bcl-2 expression and increase in Bax expression suggested that ZZCD could decrease cell death by inhibiting apoptosis (Figure 13).

As a transcription factor, CREB has been shown to be involved in cell survival (Tardito et al., 2006). Regulated by hormone signaling pathway, calcium signaling pathway, and stress-induced signaling pathway, CREB is phosphorylated and regulates the expression of BDNF, which plays a key role in neuroprotective and neurotrophic processes related to depression (Malberg and Blendy, 2005). With respect to the increase in CREB expression of ZZCD, cAMP-CREB pathway is demonstrated to be involved in cell survival and plasticity (Duman et al., 1999), and several antidepressant treatments exert up-regulation of CREB (Duman et al., 1999). Moreover, promoting cAMP-CREB pathway increases the proliferation of neural precursor cells (Nakagawa et al., 2002), which indicates that the antidepressant effects of ZZCD could be associated with neuroprotection.

### Validation of Antidepressant Effect of Zhi-zi-chi Decoction on CUMS-induced Rats

At normal conditions, a single repeated stress triggers an adaptation response. However, the chronic unpredictable mild stress can produce long-term depression in rodents, which is commonly used to establish animal models of depression (Hao et al., 2019). In this study, to ensure the efficiency and quantity of the mode of depression, nine different stresses were conducted on rats and these stimulation were not repeated every two consecutive days. According to the sub-chronic maximum tolerance dose (MTD) test of ZZ, DDC, and ZZCD that we have already performed, the MTD of ZZ, DDC, and ZZCD are 10, 15, 12 g/kg (converted into raw material) once a day for 2 weeks respectively. Therefore, the dosage and duration of ZZ, DDC, and ZZCD is determined as 5 g/kg (converted into raw material) once a day for 2 weeks. In addition, three behavioral tests were performed to evaluate the credibility of the model, including SPT, FST, and TST. Anhedonia and despair are central features of depression. Decreased sucrose solution consumption in SPT implies a defective reward system, which modeled the anhedonia (Willner et al., 1987). FST is an examination of a crucial aspect of the response to antidepressant drug action. The immobility in FST and TST is a reflection of behavioral despair (Steru et al., 1985; Petit-Demouliere et al., 2005). Thus, these tests were capable to determine the antidepressant effects of drug.

**Figure 14:**
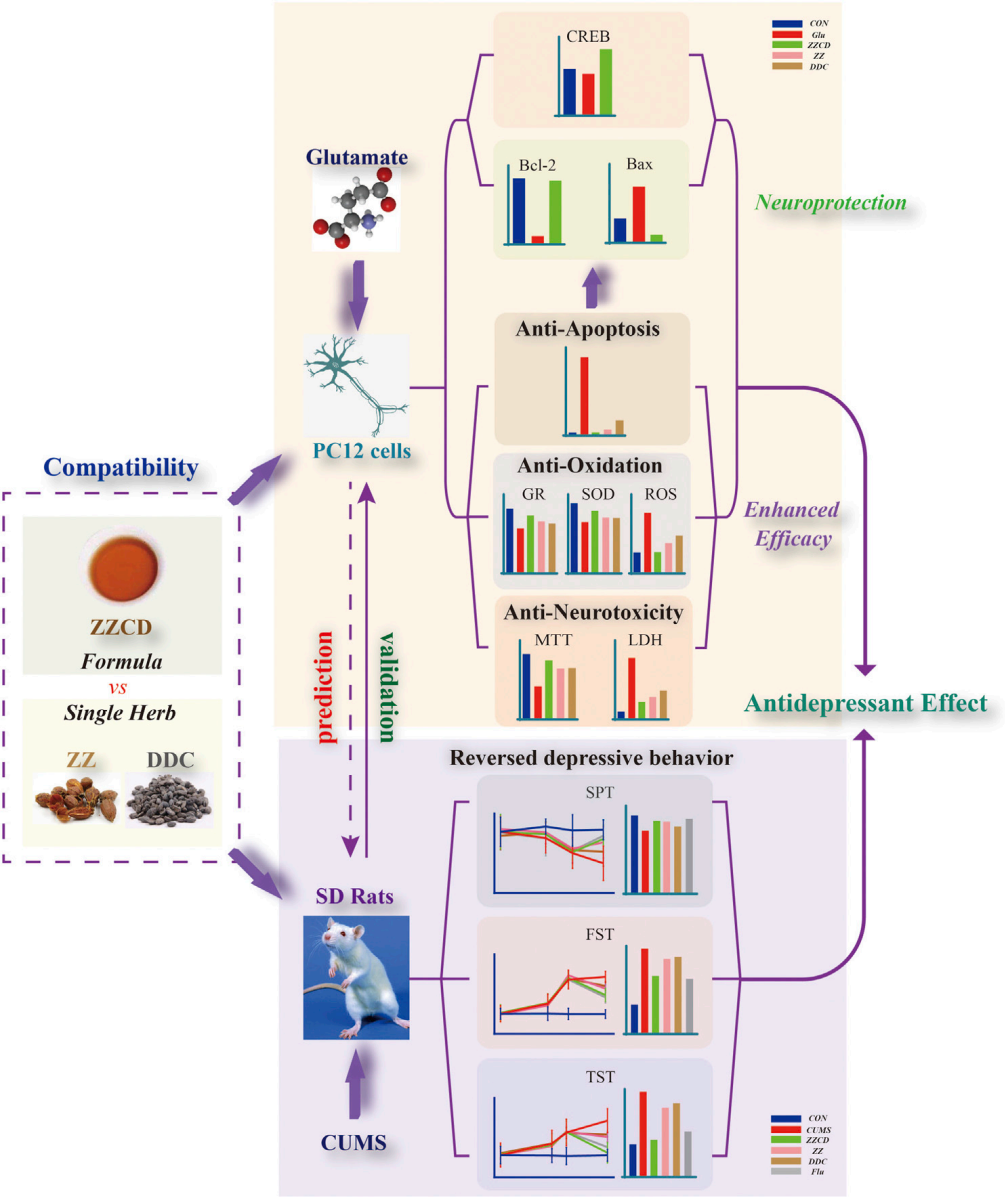
Summary of the mechanism of antidepressant effects of ZZCD.

After 5 weeks of CUMS stimulation, the sucrose preference rate was decreased sharply and the immobility time in both FST and TST were increased significantly. It suggested that CUMS induced anhedonia and despair in rats, indicating that the animal model of depression was established successfully. The interesting thing was that these phenomena was reversed by administration of ZZCD rather than ZZ or DDC for 2 weeks, which indicated that the compatibility of ZZ and DDC produces antidepressant effects by eliminating anhedonia and despair in CUMS-induced rats. Therefore, the antidepressant effects of ZZCD investigated in cell modeling was validated by the results of the animal model (Figure 14), proving that the PC12 cells treated with a high concentration of glutamate could be the optimal method for the study of antidepressant effects of other drugs. In addition, the cell model could provide as a reference for future research on the compatibility mechanism of other antidepressant traditional Chinese formulas, and even the high-throughput screening of antidepressant monomer compounds of TCM.

## Conclusion

In this study, the antidepressant effects of ZZCD were investigated from the aspect of neuroprotection in glutamate-induced PC12 cells. The compatibility of ZZ and DDC enhanced the efficacy of anti-neurotoxicity, anti-oxidation, and anti-apoptosis, which revealed that the antidepressant effects of ZZCD were closely associated with neuroprotection. In addition, the antidepressant effects of ZZCD were validated by a CUMS-induced rat modeling. Our research not only provides as a reference for future researches on the compatibility mechanism of other antidepressant traditional Chinese formulas, but also prepares for the high-throughput screening of antidepressant monomer compounds of TCM.

## Data Availability Statement

The raw data supporting the conclusions of this article will be made available by the authors, without undue reservation, to any qualified researcher.

## Ethics Statement

The animal study was reviewed and approved by Ethics Committee of the Second Military Medical University (Shanghai, China).

## Author Contributions

YZ and TZ conceived and designed the experiments. YZ wrote the manuscript. YZ, YL, DZ, and BP conducted the experiments and analyzed data. YL, JW, and TZ reviewed and revised the manuscript.

## Funding

This work was supported by National Natural Science Foundation of China (81973457, 81773862, and 81573584).

## Conflict of Interest

The authors declare that the research was conducted in the absence of any commercial or financial relationships that could be construed as a potential conflict of interest.

## Abbreviations

ANOVA, One-way analysis of variance; BCA, Bicinchoninic acid; cAMP, cyclic adenosine monophosphate; CMC-Na, Sodium carboxymethyl cellulose; CON, control; CREB, cyclic adenosine monophosphate-response element binding protein; CUMS, chronic unpredictable mild stress; DDC, Semen sojae praeparatum; DMSO, Dimethylsulfoxide; FBS, fetal bovine serum; Flu, Fluoxetine; FST, forced swim test; Glu, Glutamate; GR, Glutathione reductase; HPLC, high performance liquid chromatography; LDH, Lactate dehydrogenase; MTD, maximum tolerance dose; MTT, 3-(4,5-dimethyl-thiahiazol-2-thiazolyl)-2,5-diphenyl-2H-tetraxoliumbromide; OD, optical density; PD, pharmacodynamics; PI, Propidium iodide; ROS, reactive oxygen species; SOD, Superoxide dismutase; SPT, sucrose preference test; TCM, traditional Chinese medicine; TST, tail suspension test; UPLC-Q-TOF/MS, ultra high performance liquid chromatography-quadrupole-time of flight/mass spectrometry; ZZ, Fructus Gardeniae; ZZCD, Zhi-zi-chi decoction.
